# Replication Fork Protection Factors Controlling R-Loop Bypass and Suppression

**DOI:** 10.3390/genes8010033

**Published:** 2017-01-14

**Authors:** Emily Yun-Chia Chang, Peter C. Stirling

**Affiliations:** Terry Fox Laboratory, British Columbia Cancer Agency, 675 West 10th Ave., Vancouver, BC V5Z1L3, Canada; echang@bccrc.ca

**Keywords:** R-loop, DNA replication stress, DNA repair, transcription–replication conflict

## Abstract

Replication–transcription conflicts have been a well-studied source of genome instability for many years and have frequently been linked to defects in RNA processing. However, recent characterization of replication fork-associated proteins has revealed that defects in fork protection can directly or indirectly stabilize R-loop structures in the genome and promote transcription–replication conflicts that lead to genome instability. Defects in essential DNA replication-associated activities like topoisomerase, or the minichromosome maintenance (MCM) helicase complex, as well as fork-associated protection factors like the Fanconi anemia pathway, both appear to mitigate transcription–replication conflicts. Here, we will highlight recent advances that support the concept that normal and robust replisome function itself is a key component of mitigating R-loop coupled genome instability.

## 1. Replication Fork Protection and Genome Instability

DNA replication and transcription are essential cellular processes mediated by complex machineries that copy genetic information into complementary DNA and RNA molecules, respectively. Although both transcription and replication are accomplished by unwinding the two strands of the DNA double helix, the transcription machinery—including RNA polymerases and other transcription factors—only transcribe one DNA strand. For replication, DNA replication machineries move in opposite directions from the origin and duplicate both DNA strands, forming the replication fork structure [1]. The DNA replication machinery, called the replisome, is a macromolecular complex where the core components include a helicase that unwinds DNA, primase that facilitates the initiation of DNA synthesis, and DNA polymerase that synthesize both DNA strands as well as associated processivity factors, factors to mitigate topological constraints, process Okazaki fragments, and more [2]. As the replication fork proceeds, it can encounter obstacles causing fork progression to slow down or stop. Stalled replication forks can be major threats to the genome as, if they persist, they can lead to DNA damage and potentially to deleterious mutations. As such, cells have evolved a robust DNA replication stress response that signals to proteins able to promote replication restart, fork remodeling, or even firing of additional replication origins [3]. One major physiological obstacle to replication is the transcription machinery, since DNA replication and transcription compete for the same DNA template. Encounters or conflicts between replication and transcription are unavoidable; thus, both processes must be highly coordinated to prevent their clashes from damaging DNA, and cells have evolved strategies to resolve such conflicts, when they do occur, to preserve genome integrity [4,5].

While the spectrum of factors that regulate replication–transcription collisions and their mechanisms of action are not completely known, there have been a growing number of studies in recent years describing replication fork-associated proteins whose disruption impacts the resolution of transcription–replication conflicts. Here, we discuss the recent data that together suggest a potentially generalizable role for robust fork protection as a means to clear transcription-associated impediments to fork progression.

## 2. Replication–Transcription Conflicts and R-Loops

**Figure 1:**
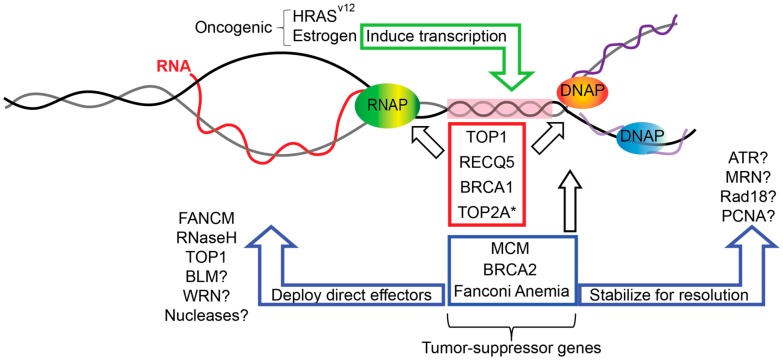
DNA replication fork protection factors mitigating transcription–replication conflicts. A nascent RNA (red) emerging from RNA polymerase (RNAP) annealed to its template could impede an incoming DNA replication fork (orange and blue polymerases (DNAP), right) to create a region of topological conflict (pink box, a replication–transcription collision). Fork protection factors with dual roles in transcription and replication (red box) or with roles at replication forks only (blue box) could act to suppress such conflicts either directly, as is the case for Fanconi anemia complementation group M (FANCM), RNaseH, and topoisomerase I (TOP1), or could act indirectly by stabilizing replication forks to allow time for canonical R-loop resolution mechanisms to act. Tumor suppressor gene mutations impact these fork protection mechanisms. Oncogenic mutations or stimuli also contribute to transcription-replication conflicts by inducing or creating aberrant transcriptional programs. * TOP2A was implicated indirectly in the cited study showing that p53 mutations increase transcription-replication conflicts [57].

Replication forks can encounter many obstacles in the genome, including DNA binding proteins or regions of DNA secondary structure [3]. One major transcription-associated structure that impairs fork progression are R-loops. R-loops are stable, three-stranded nucleic acid structures, where an extended DNA:RNA hybrid forms after transcription on the template strand and exposes the non-template DNA strand as a loop of single-stranded DNA (ssDNA). The programmed formation of R-loops contributes beneficially to important cellular processes including efficient transcription termination, mitochondrial DNA replication, immunoglobulin class switching, and epigenetic modifications [5,6,7]. Indeed, recent sequencing efforts indicate that in unperturbed cells, R-loops occupy up to 5% of the genome, and have lifetimes in the order of 10–20 min [8,9]. R-loops form at highly transcribed GC-rich sequences and have been associated with CpG island promoters in mammals [7,8]. This association with GC content is driven by a sequence property called GC-skew, in which the non-template strand is G-rich, leaving a C-rich template to pair with G-rich RNA in an R-loop. The DNA:RNA CG base-pair is thermodynamically favored and may stabilize R-loops in these regions and other regions with GC-skew. Interestingly, analysis of R-loops in the yeast genome, which lacks CpG islands and has a lower GC content, identified long homopolymeric AT base-pairs as significantly associated with R-loop formation [9,10]. We know that many other factors aside from thermodynamics control R-loop occupancy in the genome as part of normal biology. However, if R-loops are deregulated or if aberrant R-loops are removed inefficiently, this can be a major source of genome instability in cells that are unable to properly replicate through R-loop-containing regions [5,11,12]. Such collisions can occur either as head-on or codirectional collisions, of which head-on collisions are known to be more damaging (Figure 1 illustrates a head-on collision, see also [13]).

R-loops threaten genome integrity in at least two ways. The exposed ssDNA is more chemically labile and can be targeted inappropriately by DNA-modifying enzymes or repair factors, leading to mutagenesis and DNA damage [14,15]. Alternatively, R-loops can impair replication fork progression, leading to DNA replication stress, which in turn contributes to DNA double-strand break formation through fork collapse or under-replicated DNA entering mitosis [16,17]. To counteract the effects of deregulated R-loops, the most prominent and direct regulators of R-loops are RNaseH1 and RNaseH2 enzymes that specifically degrade the RNA moiety of R-loops and some helicases, such as senataxin, Aquarius, Dhx9, and Pif1 [15,18,19,20,21]. Historically, early research focused on defects in RNA-processing factors as causes of R-loops. Inactivation of RNA splicing factors such as alternative splicing factor/pre-mRNA-splicing factor SF2 (ASF/SF2), defects in the THO/TREX complex (which functions in mRNA-processing and export), and mutations in different RNA biogenesis factors have all been shown to induce R-loop-associated genome instability [22,23,24,25,26]. Work primarily conducted over the past 5 years has demonstrated that many additional proteins, not strictly involved in RNA processing, also help to clear R-loops and prevent transcription-coupled DNA damage, including many DNA repair proteins (reviewed in [27]). Many of these new factors are associated with preventing or handling replication stress, suggesting that the evolution of the replication stress response may have been coupled to activities that can promote bypass or clearance of R-loops.

## 3. Roles of Replication Fork Proteins in Bypassing or Suppressing R-Loops

Literature linking replication fork proteins to R-loops reveals relatively few core replication factors and many more fork-associated proteins, some of which associate with forks constitutively at all loci, while others are specifically required when encountering an obstacle to replication.

### 3.1. Topoisomerase I

Topoisomerase I (TOP1) was one of the first proteins with normal functions in DNA repair and transcription that was conclusively linked to the suppression of R-loop-mediated genome instability. TOP1 relieves torsional stress in DNA to facilitate transcription and DNA replication. Tuduri et al. showed that depletion of TOP1 led to genome instability and reduced replication fork speed as well as increased fork pauses or stalls [28]. These phenotypes were suppressed by overexpression of RNaseH1, which degrades RNA in R-loops, or by inhibiting transcription. Finally, they showed that DNA damage markers such as γ-H2Ax preferentially accumulated at highly transcribed genes in cells lacking TOP1 [28]. This work was followed by analysis of TOP1 function in yeast, which linked loss of *TOP1* to the accumulation of R-loops at the ribosomal DNA (rDNA) locus [29]. Using chromatin immunoprecipitation, electron microscopy, and other assays, these authors demonstrated that, in the absence of *TOP1*, R-loops accumulated over the 5′ region and the intergenic spacer sequence region of the rRNA locus. These authors proposed a model in which positive supercoiling builds up the 3′ end of the RNA polymerase in the absence of *TOP1*, leading to polymerase stalling. At the same time, negative supercoiling builds up behind RNA polymerase, facilitating strand invasion by the nascent unprocessed rRNA [29]. Work in other systems have suggested that the accumulation of supercoiling is not the only mechanism by which *TOP1* depletion could lead to R-loop-mediated DNA damage. For example, work in mammalian cells suggests a role for *TOP1* in mRNA packaging or splicing through the known R-loop regulator ASF/SF2 [28]. While there are several potential mechanisms, since these early studies, TOP1 inhibition has been frequently used to induce R-loops for studies on antisense transcription, DNA repair, and other genomic features [15,30,31].

### 3.2. Minichromosome Maintenance (MCM) Helicase Complex

One of the most recently discovered replisome components that plays a role in mitigating R-loop-induced genome instability is the core replicative helicase complex MCM2–7 [32]. Besides the primary activity of unwinding double-stranded DNA at the replication fork, the MCM2–7 complex also interacts with DNA replication checkpoint (DRC) signaling proteins and is required for proper DRC activation upon stress [2,33]. Previously, it was shown that the *mcm2DENQ* mutant, which causes the loss of a specific regulatory site of MCM2, blocked DRC signaling under stress without affecting DNA unwinding processes [33]. Further investigation revealed that the DNA damage induced in *mcm2DENQ* mutant was due to replication–transcription collisions induced by R-loop formation. The authors showed that the observed elevated R-loop structures and the DNA damage phenotypes could both be rescued by RNaseH or TOP1 overexpression, confirming the role of the MCM complex in fork protection from aberrant R-loops [32]. Interestingly, the authors circumstantially link the *mcm2DENQ* phenotypes to those of a specific *MEC1* (yeast ataxia telangiectasia and Rad3 related (ATR)) allele based on the role of ATR in replication stress signaling and the phenotypic similarity with the *mec1-4* allele. While there are no concrete links of Mec1/ATR to R-loop mitigation to our knowledge, we speculate that the role of ATR in the replication checkpoint could help reduce R-loop-associated DNA damage. Finally, replicative helicases from all three domains of life, including MCM, have been shown to displace RNA from DNA:RNA hybrid structures in vitro [34]. This raises the possibility that under some circumstances the MCM helicase activity could be sufficient to clear R-loops in vivo as part of its normal role in replisome progression.

### 3.3. RECQ5 Helicase

RECQ5 helicase is a RNA polymerase II (RNAPII)-interacting protein important for controlling the movement of RNAPII across genes and thus ensures proper transcription elongation. Loss of RECQ5 induces genome instability arising from transcriptional stress [35,36]. Besides its crucial role in transcription, RECQ5 also plays a role in DNA replication. It has been shown that RECQ5 localizes to stalled replication forks and interact directly with proliferating cell nuclear antigen (PCNA), an important component of the replisome [37]. Furthermore, deletion of RECQ5 in mouse embryonic stem cells and mouse embryonic fibroblasts induced sensitivity towards camptothecin, a potent TOP1 inhibitor that blocks replication, as well as cell death [38]. These findings imply that RECQ5 may protect genome integrity at sites of replication–transcription interference. As TOP1 resolves R-loop-induced DNA damage at stalled forks, RECQ5 could also play a part in R-loop resolution. Most recently, Li and colleagues showed that RECQ5 indirectly regulates TOP1 SUMOylation, and that this is required for reducing R-loop formation [39]. Using chromatin fractionation and separating open chromatin and heterochromatin with low transcriptional activity, the authors first showed that TOP1 is SUMOylated at K391 and K236 residues by PIAS1 E3 ligase and its cofactor serine- and arginine-rich splicing factor 1 (SRSF1) during transcription. Depletion of RECQ5 abolished TOP1 SUMOylation, weakening the interactions between TOP1 and RNAPII transcription machinery. Lastly, the authors observed accumulation of R-loops in RECQ5-depleted cells, and that the level of DNA double-strand breaks (DSBs) in RECQ5-depleted cells is reduced when overexpressing RNaseH. Interestingly, the helicase domain of RECQ5, but not the helicase activity, is required for R-loop suppression. The authors proposed that the N-terminal helicase domain of RECQ5 is responsible for the interactions with TOP1, SRSF1, and PIAS1, and that these interactions are key to preventing R-loop-mediated genome instability [39]. Following this work, another group recently provided evidence that RECQ5 resolves replication and transcription collisions in human HEK293 and U2OS cells [40]. This study indicated that RECQ5 localizes with transcription machinery in replication foci, and that depleting RECQ5 induced replication intermediates and replication fork stalling in actively transcribed genes. By directly interacting with PCNA, the authors further showed that RECQ5 is required for Rad18-dependent PCNA ubiquitination at sites of replication–transcription interference [40]. Thus, RECQ5 may contribute to suppressing transcription–replication conflicts both through its effects on TOP1 and through interactions with PCNA. How these activities are coordinated remains to be determined.

### 3.4. BRCA1 and BRCA2

Independent work on the breast cancer susceptibility genes BRCA1 and BRCA2 have also linked their functions to R-loops. BRCA1 and BRCA2 are both known to stabilize DNA replication forks and promote replication through difficult-to-replicate regions like telomeres [41,42]. Using a proximity ligation assay, Bhatia and colleagues showed that PCID2 (a subunit of the RNA export TREX complex) and BRCA2 interacted in cells [43]. BRCA2 is known to function both at sites of DNA double-strand breaks to recruit Rad51 onto ssDNA and at replication forks under stress in the Fanconi anemia pathway [44]. The connections to both replication and RNA processing prompted an investigation of R-loops in BRCA2-deficient cells. Using RNA interference (RNAi) technology as well as a hybrid-binding domain of RNaseH fused to GFP, the authors identified R-loop induction in BRCA2-depleted cells and showed that DNA:RNA hybrids accumulated in actively transcribed genes in these cells. Importantly, as BRCA2 is a tumor suppressor, replication stress in BRCA2-defective pancreatic adenocarcinoma CAPAN-1 cells was alleviated by overexpression of RNaseH1 [43].

BRCA1 has a longer standing and better defined relationship to transcription than BRCA2 [45]. In addition, further exploration of this relationship has directly linked BRCA1 recruitment to R-loops at sites of transcription termination [46]. In this context, BRCA1 was linked to a binding partner, senataxin, which is an RNA:DNA helicase with a known role in mitigating transcription–replication collisions [4,46]. Using immunoprecipitation, these authors established a role for BRCA1 at sites of senataxin-mediated transcription termination in suppressing R-loop formation and the concomitant DNA damage. In addition, this study identified a significant enrichment of mutations at these sites in the genomes of cancers lacking BRCA1 [46]. This provides evidence that R-loop-mediated genome instability may actually contribute to mutagenesis in cancer genomes.

## 4. Fanconi Anemia Pathway

The discovery of BRCA1 and BRCA2 in mitigating R-loop-induced DNA damage was followed by further investigation on the role of the Fanconi anemia (FA) pathway in R-loop resolution. The FA pathway is activated by replication stress and plays a critical role in interstrand crosslink repair [47]. BRCA1 and BRCA2 are bona fide FA genes, FANCS and FANCD1, respectively, which can be found mutated in patients with FA [47,48,49,50]. Two papers recently showed that FA pathway is crucial in controlling defects arising from aberrant R-loops. Schwab and colleagues first showed that defects in the FA pathway not only induced DNA breaks from transcription-replication conflicts, but also that these transcription-replication conflicts were caused by the accumulation of unscheduled R-loops that could be resolved by FA complementation group M (FANCM) translocase activity [51]. The central regulatory event in the FA pathway is the monoubiquitination and activation of FANCD2 and FANCI by the FA core complex, including the ATPase FANCM that possess DNA translocase activity, at stalled replication forks [49]. The authors first showed that FANCD2 depletion induced R-loop formation. Depleting FANCA, which is required for FANCD2 ubiquitination, also induced R-loop formation. These findings suggest that FA pathway activation is required to limit R-loop formation. The authors went on to demonstrate that the impaired replication progression and induced DNA damage after FANCD2 depletion could be rescued by transcription inhibition or RNaseH1 overexpression. In addition, not only did depletion of the ATPase FAMCM also induced R-loops, but purified FANCM could unwind DNA:RNA hybrids in vitro, and only the wild-type—but not the translocase-dead mutant FANCM—could disrupt DNA:RNA hybrids [51].

The role of the FA pathway in suppressing R-loop-induced replication forks stalling and DNA damage was further confirmed in a parallel study [52]. This study indicated significant accumulation of R-loops in representative genes prone to form R-loops by DNA:RNA immunoprecipitation and quantitative PCR (DRIP-qPCR) in different cell lines, including human FANCA^−/−^ and FANCD2^−/−^ FA patient cells, HeLa cells depleted of FANCD2, and primary bone marrow cells from FANCD2-deficient mice. DRIP-qPCR exploits a specific DNA:RNA hybrid antibody to purify R-loop-associated DNA from cells, which can then be quantified by qPCR or other methods. Similar to Schwab and colleagues’ findings, the authors also showed that DNA breaks are transcription dependent and that overexpressing RNaseH1 could rescue the induced DNA damage in FANCD2-depleted cells. Both groups provided evidence that defects in the FA pathway induce replication fork stalling and DNA breaks caused by cotranscriptionally formed R-loops [52]. Remarkably, one group found that formaldehyde treatment further induced R-loops in FANCD2-deficient cells [51]. Since aldehydes have recently been implicated in the pathology of FA, aldehyde toxicity-induced R-loops could be a potential driver of genome instability in FA. The implications of this work was further exemplified by a recent paper showing that FANCD2 is important for faithful DNA replication through common fragile sites [16]. Here again, the accumulation of R-loops at certain fragile sites in FANCD2-deficient cells was revealed to be a critical driver of genome instability. Further investigation will elucidate if and how the FA pathway proteins act directly on R-loops (such as FANCM) or contribute to mitigating transcription-replication conflicts through their associations at the replication fork, or by their functions in DNA repair.

## 5. Speculating on Additional Fork-Associated R-Loop Regulators

Dozens of proteins are involved in DNA replication fork protection or in the prevention of transcription–replication conflicts. Indeed, some of these proteins—such as senataxin, the FACT chromatin remodeling complex, and Mrc1—have been sufficiently reviewed elsewhere [4,5,53,54]. It is tempting to speculate that other proteins currently known to function in fork protection but not yet implicated in R-loop mitigation will emerge as R-loop regulators in the coming years (Figure 1).

For example, the role of ATR in mitigating R-loops has not been thoroughly addressed in the literature, to our knowledge. However, as noted above, yeast Mec1/ATR mutants resemble the phenotypes of R-loop-causing *mcm2DENQ* alleles [32]. In addition, ATR is known to regulate gene tethering to nuclear pores in yeast as a means to prevent topological tension during replication stress [55]. Moreover, ATR is activated by the exposure of replication protein A (RPA)-bound ssDNA [3], which could occur at an R-loop-associated replication fork, or within the R-loop itself. Indeed, signaling through a related kinase, ataxia-telangiectasia mutated (ATM), was recently shown to be influenced by R-loop formation in response to UV-induced DNA damage [56]. Thus, a potential role for ATR in mitigating transcription-replication conflicts likely has many facets and extends beyond that of the *mcm2DENQ* allele.

Another candidate is the Bloom’s syndrome helicase (BLM) and its yeast orthologue Sgs1. BLM is another RECQ-like helicase and is biochemically capable of resolving DNA:RNA hybrids in vitro [58]. Analysis of the yeast orthologue has linked it to transcription-associated genome instability [30]. Furthermore, it is known to promote both replication and transcription through the rDNA, a hotspot for R-loop formation [29,59]. Finally, BLM interacts physically and functionally with components of the FA pathway, and Bloom’s syndrome is clinical related to FA [49,60].

The role of many other factors in preventing deleterious replication R-loop encounters is unknown, but there is no shortage of candidates. The Mre11-Rad50-Nbs1 complex has functions at DNA replication forks that could impact R-loop stability or resolution [61]. The proliferating cell nuclear antigen (PCNA) processivity factor is a critical regulator of the response to replication impediments and coordinates the FA pathway through the action of the Rad18 E3 ubiquitin ligase [62]. The Werner’s syndrome helicase WRN also functions at replication forks [63]. Nucleases like Mus81 also process stalled replication forks and have been linked to common fragile sites, which are sites of FA pathway action [16,64]. Disrupting any of these factors could impact the ability of replication forks to bypass and clear R-loops, potentially stabilizing the R-loop and creating transcription-associated DNA damage. Determining whether these effects exist and which are direct versus indirect remains an active area of investigation (Figure 1).

## 6. Replication-Transcription Collisions in Cancer Genome Instability

Despite our growing appreciation for R-loops as a direct mechanism of genome instability, there are relatively few pieces of data that conclusively link R-loops to genome instability during oncogenesis in humans. It is now clear that cancer predisposition factors such as BRCA1/2 and the FA pathway do mitigate transcription–replication conflicts at R-loops to promote genome maintenance in cells. Therefore, one can imagine that transcription-mediated genome instability events could play some role in the mutagenesis that gives rise to cancer in people bearing germline defects in these DNA repair pathways. As noted above, mutation signatures in BRCA1-deficient tumors matched sites of high R-loop formation in cell lines [46]. Indeed, recent work has shown that the aberrant transcriptional program induced in cells expressing the HRAS^v12^ oncogene was sufficient to cause an increase in unscheduled R-loops that drove DNA replication stress [65]. In addition, p53 has been ascribed a new role in DNA replication stress and linked to increased transcription–replication conflicts through topological constraints that may involve R-loops [57,66]. As surprising were recent findings that estrogen-induced DNA damage occurs by stimulating a transcriptional program that favors R-loop formation and associated replication-dependent DNA damage [67]. Thus, there are compelling preclinical links of R-loop formation to genome instability in cancer (Figure 1). If and how this information will be translated into studies of human cancer predisposition, used as a biomarker for ongoing replication stress, or serve as a phenotype that can be leveraged therapeutically, is an exciting prospect for research on R-loops and replication fork protection in the coming years. Indeed, this work will likely extend beyond cancer research, as R-loops are now implicated in various neurodegenerative, autoimmune, and developmental disorders (reviewed in [68]).

## 7. Conclusions

Research, especially in recent years, has shown R-loops as important modulators of genome integrity and dynamics as well as its implications in various human diseases. From RNA processing factors and tumor suppressors to the more recently discovered replication fork-associated proteins, our knowledge on factors important to R-loop homeostasis that prevent and counteract deleterious outcomes caused by deregulated R-loops has grown immensely. R-loop biology remains an active area of research and uncovering detailed mechanisms controlling R-loops is crucial to understanding human diseases and identifying novel therapeutic strategies in the future.
